# Association of Galanin and Major Depressive Disorder in the Chinese Han Population

**DOI:** 10.1371/journal.pone.0064617

**Published:** 2013-05-31

**Authors:** Yong-Jun Wang, Hui Li, Yu-Tao Yang, Chang-Le Tie, Feng Li, Zhi-Qing David Xu, Chuan-Yue Wang

**Affiliations:** 1 Tianjin Anding Hospital, Tianjin, China; 2 Department of Neurobiology, Beijing Key Laboratory of Major Brain Disorders, Beijing Institute for Brain Disorders, Capital Medical University, Beijing, China; 3 Beijing Anding Hospital, Capital Medical University, Beijing, China; Baylor College of Medicine, United States of America

## Abstract

**Objective:**

This study aimed to investigate the association of galanin (GAL) gene and the development of depression in the Chinese Han population.

**Methods:**

A total of 700 patients with depression who met the diagnostic criteria of Diagnostic and Statistical Manual of Mental Disorders, Fourth Edition (DSM-IV) and 673 healthy controls were used in this study. Ligase detection reactions were performed on 10 selected single nucleotide polymorphism (SNP) sites in the GAL gene. A series of statistical methods were carried out to investigate the correlation between the GAL gene SNP and the patient susceptibility to depression.

**Results:**

The SNPs of rs694066 in the GAL gene showed a positive correlation with MDD. Compared with the healthy controls, lower frequency of G/G genotype and higher frequency of A/G genotype were observed in rs694066 in MDD patients, a lower frequency of G-allele and higher frequency of A-allele were observed in rs694066. These correlations were more pronounced in the 376 female patients and 360 female control subjects than in the 324 male patients and 313 healthy male subjects.

**Conclusions:**

This study investigated the relationship between the GAL gene SNP and the susceptibility to depression in the Chinese Han population. The findings clearly indicate that the GAL gene polymorphism is closely correlated to the incidence of depression in the Chinese Han female patients.

## Introduction

Major depressive disorder (MDD) is a common and debilitating condition with pervasive impact on the quality of life for both patients and their families [1]. It is estimated that 10% to 15% of the general population experience clinical depression during their lifetime. MDD is twice as common in women as in men [2], [3] and is associated with a high morbidity rate [4], thereby constituting a significant worldwide health burden. The etiology of MDD is not fully understood now. It is generally recognized that genetic, environmental, psychological and social factors all contribute to the pathogenesis of depression[5], [6]. Family, twin, and adoption studies indicate that genetic factors play a particularly important role in the development of MDD[7], [8]. Twin studies suggested that the heritability of MDD is 40% to 50%, while family studies indicate that the first-degree relatives of MDD patients have a two to three-fold increase in the lifetime risk of developing the disorder [9]. These studies make it possible to identify genes of substantial influence on MDD risk through molecular genetic techniques. Both animal and human studies have demonstrated that the neuropeptide galanin (GAL), which widely expressed in the brain, spinal cord and gut, possesses a potent antidepressant activity [10]. This implies that the GAL gene may be involved in the development of MDD.

This study aimed to investigate the association of single nucleotide polymorphism (SNP) of the GAL gene with the risk of developing MDD in the Chinese Han population.

## Materials and Methods

### Ethics Statement

This study was approved by the Research Ethics Committee of the Capital Medical University Hospital and written informed consent was obtained from each of the participants or guardians. The objective and procedures of this study were explained to all of the subjects and the patient’s guardians. Healthy control subjects and some patients who could read the consent signed the informed consent forms themselves. Some patients’ guardians consented to sign the informed consent forms on behalf of the participants whose capacity to consent was compromised. All potential participants who declined to participate or otherwise did not participate were eligible for treatment and were not disadvantaged in any other way by not participating in the study.

### Subjects

Seven hundred patients (324 men and 376 women; mean age 40±14.9 years) diagnosed with MDD by independent physicians blind to the study design using the criteria of Diagnostic and Statistical Manual of Mental Disorders, Fourth Edition (DSM-IV) were recruited. Six hundred and seventy-three healthy volunteers (313 men and 360 women; mean age 41.9±17.2 years) were included as controls. All subjects were screened by two trained psychiatrists using the DSM-IV Axis I disorder (SCID-I/P). Sex was not limited and 18< age <60. MDD patients had 2 or more times of depressive episode and without Electroconvulsive therapy (ECT) in half a year and without the use of antidepressants treatment in 1 month. Healthy controls had no mental disease and family history and HAMD-17≤7. Patients who had a history of alcohol or drug dependence, or who had a full-scale IQ of less than 80 measured by the Wechsler Intelligence Scale were excluded. The onset age of patients was 30.36±12.44 years, the duration of illness were 54.20±82.84 months and the average years of education were 12.04±3.04 years.

### DNA Isolation and Amplification

Genomic DNA was extracted from whole blood samples using a genomic DNA Purification Kit (TAKARA). Genotyping for GAL polymorphisms was conducted by polymerase chain reaction (PCR) followed by ligase detection reaction (LDR). Fragments of the GAL gene, each harboring one of the SNP sites listed in table S1, were amplified by a series of PCR reactions. All PCR primers and gene probes used in LDR were designed using the Primer 5.0 and Oligo 6.0 software and synthesized by Shanghai Biowing Applied Biotechnology Company. All PCR reactions were performed at 94°C for 2 min, followed by 35 cycles (30s at 94°C, 20s at 59°C, 62°C or 53°C, and 60s at 72°C), and a final extension at 72°C for 3 min.

### Ligation Detection Reaction

LDRs were performed on a MJ PCT-200 Gradient thermal cycler in 20 μL of buffer containing 20 mM Tris–HCl (pH 7.6), 10 mM MgCl_2_, 100 mM KCl, 10 mM DTT, 1 mM EDTA, 1mM NAD^+^, 12.5 nM of each probe, 1 μL PCR product, and 0.1 mM DNA ligase. The reaction included an initial incubation at 95°C for 2 min and then cycling for 35 cycles at 94°C for 30 s and 60°C for 2 min. After addition of 0.5 mL 0.5 mM EDTA to stop the reaction, the products (1 μL) were mixed with an equal volume of ABI GS-500 ROX (a fluorescent labeled molecular weight standard) and deionized formamide. The mixture was denatured at 95°C for 2 min, chilled on ice rapidly, loaded on a 5% polyacrylamide and 5 M urea gel, and electrophoresed for 2.5 h at 3000V (377 DNA Prism Sequencer; Applied Biosystems). Genemapper software was used to analyze the ligation products.

### Statistical Analysis

All analyses were performed using SPSS software program Version 16.0 for Windows (SPSS Institute Inc., Chicago, IL, USA). Chi-square test was used to analyze the differences in genotypic frequency distributions of GAL polymorphisms between the MDD cases and the control subjects and Hardy-Weinberg equilibrium test was performed to determine the departure. The polymorphism data were stratified on the basis of sex. Power analyses were completed using the Genetics Power Calculator (http://pngu.mgh.harvard.edu/~purcell/gpc/). The association between the combined genotypes and the haplotypes of GAL polymorphisms was evaluated by multivariate logistic regression. All tests were two-sided and differences were considered statistically significant when *P*<0.05.

## Results

### SNPs

According to the results of the test, all alleles and genotypic of the selected ten GAL SNPs were in Hardy-Weinberg equilibrium (*P*>0.05) (table S2).

### Power Analyses

The power for the overall samples was 92.23% with the genotype relative risk of 2.2 at a nominal *P* = 0.05 for minor alllele frequency of 0.13. After the samples were stratified by gender, the power for female and male was estimated at 86.65% and 82.96% respectively.

### Allele and Genotypic Frequency Distribution

The relationship between GAL SNPs and the susceptibility of MDD were analyzed (Table 1 and Table 2). The genotype of rs694066 might be correlated with MDD. Compared with healthy control subjects, MDD patients had a lower frequency of G/G genotype in rs694066 and a higher frequent of A/G genotype (*χ2* = 15.911, *P* = 0.0000673), a lower frequency of G-allele in rs694066 and a higher A-allele (*OR* = 2.216, 95%CI = 1.472–3.337, *χ2* = 15.216, *P* = 0.000097). After Bonferroni correction and permutation test (10,000 times), the above differences were still significant between patients and control subjects (*P* = 0.0018 and *P* = 0.01, respectively).

**Table 1:** The correlation analysis between the Alleles of GAL SNPs and the susceptibility of MDD.

SNPs	Group	Allele1 (freq)	Allele2 (freq)	OR	95%CI	?^2^	*P*
rs2510387	MDD	A (0.867)	G (0.133)	0.943	0.752∼1.183	0.255	0.613
	Con	0.874	0.126				
rs2513297	MDD	G (0.868)	A (0.132)	1.104	0.877∼1.389	0.712	0.399
	Con	0.879	0.121				
rs2187331	MDD	A (0.876)	G (0.124)	0.940	0.748∼1.182	0.279	0.5977
	Con	0.869	0.131				
rs948854	MDD	A (0.862)	G (0.138)	0.860	0.696∼1.063	1.939	0.164
	Con	0.845	0.155				
rs2097042	MDD	A (0.840)	G (0.160)	1.026	0.833∼1.264	0.058	0.809
	Con	0.843	0.157				
rs4432027	MDD	T (0.854)	C (0.146)	1.149	0.930∼1.418	1.662	0.197
	Con	0.836	0.164				
rs694066	MDD	G (0.944)	A (0.056)	2.216	1.472∼3.337	15.215	9.7E-5
	Con	0.974	0.026				
rs1546309	MDD	T (0.835)	C (0.165)	1.141	0.932∼1.395	1.637	0.201
	Con	0.816	0.184				
rs3136540	MDD	C (0.846)	T (0.154)	0.839	0.683∼1.031	2.798	0.094
	Con	0.822	0.178				
rs1042577	MDD	C (0.786)	T (0.214)	0.936	0.779∼1.124	0.595	0.440
	Con	0.774	0.226				

**Table 2:** The correlation analysis between the Genotype of GAL SNPs and the susceptibility of MDD.

SNPs	Group	Genotype1 (freq)	Genotype2 (freq)	Genotype3 (freq)	?^2^	*P*
rs2510387	MDD	A/A (0.747)	A/G (0.242)	G/G(0.012)	0.632	0.729
	Con	0.762	0.224	0.014		
rs2513297	MDD	G/G(0.754)	A/G (0.228)	A/A (0.018)	1.074	0.584
	Con	0.777	0.205	0.019		
rs2187331	MDD	A/A (0.756)	A/G (0.227)	G/G (0.018)	1.144	0.565
	Con	0.773	0.205	0.022		
rs948854	MDD	A/A (0.694)	A/G (0.296)	G/G (0.010)	4.529	0.104
	Con	0.737	0.247	0.015		
rs2097042	MDD	A/A (0.705)	A/G (0.278)	G/G (0.018)	1.201	0.549
	Con	0.706	0.268	0.026		
rs4432027	MDD	T/T (0.692)	T/C (0.289)	C/C (0.019)	3.405	0.182
	Con	0.732	0.245	0.023		
rs694066	MDD	G/G (0.887)	A/G (0.113)		15.911	6.73E-005
	Con	0.947	0.053			
rs1546309	MDD	T/T (0.662)	T/C (0.308)	C/C (0.030)	1.692	0.429
	Con	0.695	0.280	0.025		
rs3136540	MDD	C/C (0.674)	C/T (0.297)	T/T (0.030)	2.930	0.231
	Con	0.713	0.267	0.020		
rs1042577	MDD	C/C (0.598)	C/T (0.354)	T/T (0.048)	1.051	0.591
	Con	0.610	0.353	0.037		

When the data on SNPs and the incidence of MDD were analyzed on the basis of gender, it turned out that the 376 female MDD patients and 360 female healthy control subjects had a significant lower frequency of genotype G/G in rs694066, a significant higher frequency of genotype A/G, a lower frequency of the G-allele and a higher frequency of A-allele in rs694066. In contrast, no SNP showed a significant correlation with MDD in the male subjects. (Table 3 and Table 4).

**Table 3:** The correlation analysis between Alleles of rs694066and the susceptibility of MDD.

sex	Group	Allele1 (freq)	Allele2 (freq)	OR	95%CI	?2	*P*	*Pc*
female (376)	MDD	G (0.938)	A (0.062)	2.647	1.505–4.656	12.243	0.0005	0.005
	Con	0.975	0.025					
male (324)	MDD	G (0.951)	A (0.049)	1.788	0.982–3.254	3.704	0.054	0.54
	Con	0.972	0.028					

*Pc*: Bonferroni adjusted *p* value.

**Table 4:** The correlation analysis between Genotypes of rs694066 and the susceptibility of MDD.

Sex	Group	Genotype1 (freq)	Genotype2 (freq)	?2	*P*	*Pc*
female (376)	MDD	G/G (0.875)	A/G (0.125)	12.838	0.0003	0.003
	Con	0.951	0.049			
male (324)	MDD	G/G (0.901)	A/G (0.099)	3.862	0.050	0.50
	Con	0.944	0.056			

*Pc*: Bonferroni adjusted *p* value.

### Linkage Disequilibrium and Haplotype Incidence

Linkage disequilibrium analysis was performed on the 10 identified GAL SNPs by using the Haploview software. As shown in table S3, six closely linked SNPs in GAL were detected between rs251038 and rs2513297, rs1546309 and rs3136540, rs2513297 and rs2187331, rs251038 and rs948854, rs2187331 and rs948854, rs3136540 and rs1042577 (1≥D′>0.8). Ten GAL SNPs were divided into two blocks according to the D value from linkage disequilibrium analysis, and their correlations with MDD were analyzed. The results showed that the incidence of MDD was highly correlated with haplotype A-A of rs2510387-rs2513297 (*P* = 0.030), haplotypes A-G-A-A and G-G-A-A of rs2510387-rs2513297-rs2187331-rs948854 (*P* = 0.022 and *P* = 0.028, respectively), and haplotypes A-G-A-A-A-T and G-G-A-A-A-T of rs2510387-rs2513297-rs2187331-rs948854-rs2097042-rs4432027 (*p* = 0.035 and *p* = 0.005, respectively).

## Discussion

In this study, we detected 10 SNPs in the GAL gene in 700 patients with MDD and 673 healthy controls by using ligase detection reaction. All subjects were Chinese Han. The psychiatric diagnosis was made according to the criteria of DSM-IV. Our analyses showed a significant correlation between GAL gene polymorphisms of rs694066 and the susceptibility of MDD. More interestingly, this correlation was gender-dependent: a positive correlation between GAL SNPs and the incidence of MDD was observed only in female patients but not in male patients. Recent studies have demonstrated that much more women than men suffered MDD [4], [11]. This difference may be related to the difference in GAL gene polymorphisms between male and female patients.

A large number of studies have shown that the release disturbance of 5-hydroxytryptamine (5-HT) and norepinephrine (NE) might be associated with an increased susceptibility to depression [12], [13], [14]. GAL is a neuropeptide encoded by the GAL gene, which is widely expressed in the brain, spinal cord, and gut of humans and other mammals. Both direct and indirect evidences suggest that GAL plays a regulatory role in the MDD-associated disorder of 5-HT and NE [15], [16]. GAL significantly inhibits the stimulation-evoked NE release in a dose-dependent manner [17] and exerts an inhibitory effect via increasing the K^+^ conductance in serotonergic dorsal raphe neurons and noradrenergic locus coeruleus neurons by acting on a postsynaptic receptor [18], [19]. In addition, GAL at low possibly physiological concentrations enhances the inhibitory effect of 5-HT at the cell soma level [18]. All these pieces of evidence suggest that GAL may play an important role in the pathogenesis of depression.

Similar results have been also demonstrated in two other independent studies of depression and panic disorders in Sweden, in which the GAL gene polymorphism site of rs948854 showed a high degree of correlation with the severity of symptoms of female patients with depression[20], [21]. Although different SNP sites in GAL were reported in these studies, all these observations suggest a positive correlation between the GAL gene polymorphism and the susceptibility of female patients to depression. The expression level or function of genes in different species or populations might be different. GAL receptor knockout mice showed anxiety-like behaviors, but in rats resulted in a significant antidepressant effect [22]. Therefore, species or race-associated differences may, at least partially, explain why depression is correlated with SNPs of rs694066 in GAL in Chinese Han women but with GAL SNPs of rs948854 in Swedish women [21].

In conclusion, this study has demonstrated a positive correlation between the SNP site of rs694066 in the GAL gene and the susceptibility of the female but not male Chinese Han patients to depression. We have also demonstrated that patients with MDD have a lower frequency of G-allele of rs694066 and a higher A-allele. However, there are some limitations in this study. First, the sample size is not large enough. Second, the chosen SNPs may not represent the whole gene of GAL. Therefore, further functional studies involving a larger sample size are warranted.

## Supporting Information

Table S1 Primers and Probes Used in the Polymerase Chain Reaction–Ligase Detection Reaction Protocol.
(DOC)

Table S2 GAL SNPs detection and HWE test from Chinese Han population.
(DOC)

Table S3 Haplotype correlation analysis of depression.
(DOC)
