# Supplementary material for: Association of Galanin and Major Depressive Disorder in the Chinese Han Population
Source: PLoS One. 2013 May 31;8(5):e64617. doi: 10.1371/journal.pone.0064617 (PMC3669409; doi:10.1371/journal.pone.0064617)
Supplement: Table S1 — Primers and Probes Used in the Polymerase Chain Reaction–Ligase Detection Reaction Protocol. (DOC) [file pone.0064617.s001.doc]

**Table S1. Primers and Probes Used in the Polymerase Chain Reaction–Ligase Detection Reaction Protocol**

| Primers and probes | Sequences (5’-3’) | | Length of products |
| --- | --- | --- | --- |
| PCR primers | | | |
| rs2510387 | TCTGGATCATGGTGGGTGTA | AGACGACAGCTTTCCACGAG | 226 bp |
| rs2513297 | TTCTGGAGCTGAAGGAGGAG | GAGCCTAGAAGTGCCTGTGG | 229 bp |
| rs2187331 | TTTCGAGTGCAAGCTGAGAA | TCCTCCTTCAGCTCCAGAAC | 235 bp |
| rs948854 | GACAGTGAGTCGCCATGAGA | GGGCATGAGAGGACATCATT | 122 bp |
| rs2097042 | GGGAGGGGTCTTAGGAACAG | CAGACTCTCATGGCGACTCA | 237 bp |
| rs4432027 | GACCTCCCTCCTGTTTCACT | AATGGCTGGACCTGCTTAAA | 184 bp |
| rs694066 | GTCCAGCCTCGTTTTTCCTT | TTTCGCGAGCTATCCAAAAG | 154 bp |
| rs1546309 | GGGTGCATCAGTGACATGAG | GTGCAGGAAAGGAAATGGAA | 197 bp |
| rs3136540 | GAAGCACCCTCTGCACAAGT | GGGCCTTTTACGTTTCCAGT | 188 bp |
| rs1042577 | TGCATAAATTGGCCGAAGAT | CCTGTAGCATGTGTCGTGGT | 237 bp |
| Probes | | | |
| rs2510387R_ modify | P-TGAGTTGCCGCTCGCCGTCTGCTGTTTTTTTTTTTTTTTTTTT-FAM | |  |
| rs2510387R_A | TTTTTTTTTTTTTTTTTTATTGATCTCACTTGCATAGGATGGT | | 86 bp |
| rs2510387R_G | TTTTTTTTTTTTTTTTTTTTATTGATCTCACTTGCATAGGATGGC | | 88 bp |
| rs2513297_modify | P-ACGAAAACACATGGACTTAGGGCCCTTTTTTTTTTTTTTTTTT-FAM | |  |
| rs2513297_A | TTTTTTTTTTTTTTTTTTCACATTTCACAAAAAATTTACAACT | | 86 bp |
| rs2513297_G | TTTTTTTTTTTTTTTTTTTTCACATTTCACAAAAAATTTACAACC | | 88 bp |
| rs2187331_modify | P-TTCTCCCTTCTCAGCTTGCACTCGATTTTTTTTTTTTTTTTTTTTTTTTTTTT-FAM | |  |
| rs2187331_A | TTTTTTTTTTTTTTTTTTTTTTTTTTTTCCTCCAAAACTTGCTCTTCCTCCTT | | 106 bp |
| rs2187331_G | TTTTTTTTTTTTTTTTTTTTTTTTTTTTTTCCTCCAAAACTTGCTCTTCCTCCTC | | 108 bp |
| rs948854_modify | P-GCCTCTCGGCTGTCCTTCTGCCCACTTTTTTTTTTTTTTTT-FAM | |  |
| rs948854_A | TTTTTTTTTTTTTTTTACAGGAACGTGCCCTCTGCTCCTCT | | 82 bp |
| rs948854_G | TTTTTTTTTTTTTTTTTTACAGGAACGTGCCCTCTGCTCCTCC | | 84 bp |
| rs2097042_modify | P-GAAATCAGCCTGTGAGTCCTAGTTATTTTTTTTTTTTTTTT-FAM | |  |
| rs2097042_A | TTTTTTTTTTTTTTTTCACTTCAGGGTTTACTGTGGGCTCT | | 82 bp |
| rs2097042_G | TTTTTTTTTTTTTTTTTTCACTTCAGGGTTTACTGTGGGCTCC | | 84 bp |
| rs4432027_modify | P-GCCTCTCAGTGCCTCCCCTGGCTGCTTTTTTTTTTTTTTTTTTTT-FAM | |  |
| rs4432027_C | TTTTTTTTTTTTTTTTTTTTCCACAGGCACCAGCTCAGAAGCTCG | | 90 bp |
| rs4432027_T | TTTTTTTTTTTTTTTTTTTTTTCCACAGGCACCAGCTCAGAAGCTCA | | 92 bp |
| rs694066_modify | P-GGCATGGCAGAGGACTTAGAACAATTTTTTTTTTTTTTTTTTTTTTTTTTT-FAM | |  |
| rs694066_A | TTTTTTTTTTTTTTTTTTTTTTTTTTTGAATGGGTGCACCCAGGCTTTCCT | | 102 bp |
| rs694066_G | TTTTTTTTTTTTTTTTTTTTTTTTTTTTTGAATGGGTGCACCCAGGCTTTCCC | | 104 bp |
| rs1546309_modify | P-GTGGACTGTGCCATCTACAAGGCCATTTTTTTTTTTTTTTTTT-FAM | |  |
| rs1546309_C | TTTTTTTTTTTTTTTTTTCTGTGTTTCCAGCTGGTTGGCCTGG | | 86 bp |
| rs1546309_T | TTTTTTTTTTTTTTTTTTTTCTGTGTTTCCAGCTGGTTGGCCTGA | | 88 bp |
| rs3136540_modify | P-TGATATTTCACATACCTTTGAGATGTTTTTTTTTTTTTTTT-FAM | |  |
| rs3136540_C | TTTTTTTTTTTTTTTTTACTAAGAGATGAATGTTAAGTTGG | | 82 bp |
| rs3136540_T | TTTTTTTTTTTTTTTTTTTACTAAGAGATGAATGTTAAGTTGA | | 84 bp |
| rs1042577R_modify | P-TGCCCAGGAGGCTCTCAGGACCGCTTTTTTTTTTTTTTTTTTTTT-FAM | |  |
| rs1042577R_C | TTTTTTTTTTTTTTTTTTTTTCAGGTTACAGCACACAGACAAACG | | 90 bp |
| rs1042577R_T | TTTTTTTTTTTTTTTTTTTTTTTCAGGTTACAGCACACAGACAAACA | | 92 bp |
