# Supplementary material for: Association of Galanin and Major Depressive Disorder in the Chinese Han Population
Source: PLoS One. 2013 May 31;8(5):e64617. doi: 10.1371/journal.pone.0064617 (PMC3669409; doi:10.1371/journal.pone.0064617)
Supplement: Table S2 — GAL SNPs detection and HWE test from Chinese Han population. (DOC) [file pone.0064617.s002.doc]

**Table S2. GAL SNPs detection and HWE test from Chinese Han population**

| SNP | Allele | MAF | Call Rate | O（HET） | E（HET） | p |
| --- | --- | --- | --- | --- | --- | --- |
| rs2510387 | A/G | 0.129 | 98.4% | 0.233 | 0.225 | 0.253 |
| rs2513297 | A/G | 0.127 | 97.5% | 0.217 | 0.221 | 0.535 |
| rs2187331 | A/G | 0.128 | 97.7% | 0.216 | 0.223 | 0.326 |
| rs948854 | A/G | 0.147 | 99.6% | 0.269 | 0.251 | 0.086 |
| rs2097042 | A/G | 0.158 | 98.0% | 0.273 | 0.266 | 0.454 |
| rs4432027 | T / C | 0.155 | 98.0% | 0.268 | 0.262 | 0.499 |
| rs694066 | G/A | 0.039 | 98.9% | 0.078 | 0.075 | 0.239 |
| rs1546309 | C/T | 0.175 | 97.3% | 0.294 | 0.288 | 0.499 |
| rs3136540 | C/T | 0.166 | 97.8% | 0.282 | 0.277 | 0.599 |
| rs1042577 | C/T | 0.220 | 98.2% | 0.354 | 0.343 | 0.276 |

HWE: Hardy-Weinberg equilibrium；MAF：minor allele frequency；O（HET）：observed heterozygosity ；E(HET): predicted heterozygosity.
