# Supplementary material for: Association of Galanin and Major Depressive Disorder in the Chinese Han Population
Source: PLoS One. 2013 May 31;8(5):e64617. doi: 10.1371/journal.pone.0064617 (PMC3669409; doi:10.1371/journal.pone.0064617)
Supplement: Table S3 — Haplotype correlation analysis of depression. (DOC) [file pone.0064617.s003.doc]

**Table S3. Haplotype correlation analysis of depression**

| Gene | sex | SNPs | Haplotype | Afreq | Chi | *P* |
| --- | --- | --- | --- | --- | --- | --- |
| GAL | All | rs2510387-rs2513297 | AA | 0.011 | 4.684 | 0.030 |
|  |  | rs1546309-rs3136540-  rs1042577 | TCC | 0.742 | 0.811 | 0.367 |
|  |  |  | CTT | 0.140 | 3.085 | 0.079 |
|  |  |  | TCT | 0.072 | 0.423 | 0.515 |
|  |  |  | CCC | 0.018 | 0.909 | 0.340 |
|  |  |  | CTC | 0.015 | 0.548 | 0.459 |
|  |  | rs2510387-rs2513297-  rs2187331-rs948854 | AGAA | 0.822 | 5.224 | 0.022 |
|  |  |  | GGAA | 0.012 | 4.815 | 0.028 |
|  |  |  | GAGG | 0.104 | 0.001 | 0.970 |
|  |  |  | AGAG | 0.034 | 3.446 | 0.063 |
|  |  | rs2510387-rs2513297-  rs2187331-rs948854-rs2097042-4432027 | AGAAAT | 0.772 | 4.427 | 0.035 |
|  |  |  | GGAAAT | 0.011 | 7.757 | 0.005 |
|  | Female | rs2510387-rs2513297-  rs2187331-rs948854 | AGAA | 0.823 | 7.282 | 0.007 |
|  |  |  | AGAG | 0.034 | 10.366 | 0.001 |
|  |  | rs4432027-rs694066- rs15463091-rs1042577 | CACTT | 0.028 | 7.934 | 0.005 |
